# Languages Are Still a Major Barrier to Global Science

**DOI:** 10.1371/journal.pbio.2000933

**Published:** 2016-12-29

**Authors:** Tatsuya Amano, Juan P. González-Varo, William J. Sutherland

**Affiliations:** 1 Conservation Science Group, Department of Zoology, University of Cambridge, Cambridge, United Kingdom; 2 Centre for the Study of Existential Risk, University of Cambridge, Cambridge, United Kingdom

## Abstract

While it is recognized that language can pose a barrier to the transfer of scientific knowledge, the convergence on English as the global language of science may suggest that this problem has been resolved. However, our survey searching Google Scholar in 16 languages revealed that 35.6% of 75,513 scientific documents on biodiversity conservation published in 2014 were not in English. Ignoring such non-English knowledge can cause biases in our understanding of study systems. Furthermore, as publication in English has become prevalent, scientific knowledge is often unavailable in local languages. This hinders its use by field practitioners and policy makers for local environmental issues; 54% of protected area directors in Spain identified languages as a barrier. We urge scientific communities to make a more concerted effort to tackle this problem and propose potential approaches both for compiling non-English scientific knowledge effectively and for enhancing the multilingualization of new and existing knowledge available only in English for the users of such knowledge.

English is obviously the language that currently dominates global scientific activities as a lingua franca [1]. Locally, however, many scientists and users of scientific information, such as policy makers, communicate on a daily basis in languages other than English, which inevitably creates barriers to the transfer of knowledge between communities [2, 3]. However, the magnitude of this problem is not well quantified, and the consequences and solutions deserve further exploration. Language barriers may be a particularly serious problem in subjects in which local knowledge is especially important, such as environmental sciences required for biodiversity conservation [4]. Languages can seriously limit the transfer of knowledge in environmental sciences in two directions: when compiling scientific knowledge—for example, in global assessments, such as those by the Intergovernmental Platform on Biodiversity and Ecosystem Services (IPBES)—and when applying knowledge to local environmental issues, often tackled by field practitioners and local policy makers. Focusing on environmental sciences as an example, we here investigate the potential extent and consequences of language barriers in the two directions and propose solutions for reducing this potentially overlooked problem.

Language barriers can cause gaps in information availability during the global compilation of scientific knowledge, as scientific information is available not only in English but also in many other languages. We tried to estimate the number of conservation-related scientific documents published in the world’s major languages. Searching for scientific documents published in 2014 with two keywords, “biodiversity” and “conservation”, in 16 languages on Google Scholar generated 75,513 manuscripts, of which English was by far the most frequently used language (48,600 scientific documents, 64.4%), followed by Spanish (9,520), Portuguese (7,800), simplified Chinese (4,540), and French (2,290) (Fig 1). The other 11 languages surveyed were used in a total of 2,763 documents (see S1 Table for more detail). By further investigating 95 sample documents from those obtained using Spanish terms (the sample size was validated by the “sample.size.prop” function in R package “samplingbook”, assuming the expected proportion was P = 0.48 [i.e., the actual proportion of Spanish-only documents], a finite small population correction of N = 9,520, precision e = 0.1, and confidence level = 0.95), we confirmed that all but one document was indeed written in Spanish. Furthermore, 46 (48% of the 95) of these documents provided neither the title nor the abstract in English (Fig 2A). The result was similar when we investigated 80 sample documents from those obtained using Japanese terms (the sample size was determined in the same way but with N = 474); 35% of those documents provided neither the title nor the abstract in English (Fig 3A). Assuming similar proportions apply to other languages, these results suggest: (1) most of the 35.6% scientific documents written in a non-English language cannot be understood fully without the relevant non-English language skills, and (2) up to half of the non-English scientific documents are, in theory, unsearchable using English keywords. Moreover, having English titles and abstracts may not suffice; of the 6 peer-reviewed papers published in Japanese by the first author of this paper (all with an English title and 3 also with an English abstract), 4 were not searchable using their English titles on Google Scholar, nor were two searchable on Web of Science. All 6, however, appeared on Google Scholar when searched using their Japanese titles. Google Scholar searches can include “grey literature” (usually not peer-reviewed). However, of the 46 Spanish documents with neither an English title nor an English abstract, over half (26) were journal articles, books, or theses (Fig 2B) and thus are expected to have scientific credibility. Similarly, 43% (12) of the 28 Japanese documents with neither an English title nor an English abstract were journal articles (Fig 3B). This proportion was higher in those documents with an English title and/or an English abstract in both languages (Figs 2C and 3C). While some of these non-English journals might not necessarily be committed to publishing papers of reasonable quality [5], there are also well-established journals that regularly publish a non-negligible number of peer-reviewed papers on biodiversity conservation in non-English languages (see examples in S1 Table). The same is true for scientific data: global (i.e., English-based) biodiversity databases store fewer data from countries with fewer English speakers [6], but this could be partly because data from those countries are not necessarily available in English. For example, over 4 million records on species occurrence and abundance, including over 1 million based on monitoring surveys organized by the Ministry of the Environment in Japan, are available online (http://ikilog.biodic.go.jp/) but currently only in Japanese.

**Fig 1:**
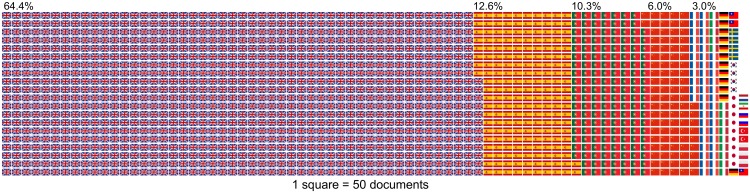
Waffle plot of the number of scientific documents in 2014 alone based on a search with two keywords—“biodiversity” and “conservation”—in 16 major languages on Google Scholar. Each square represents 50 documents. The flags merely represent the language of each document, not where the work originated. See S1 Table for more detail.

**Fig 2:**
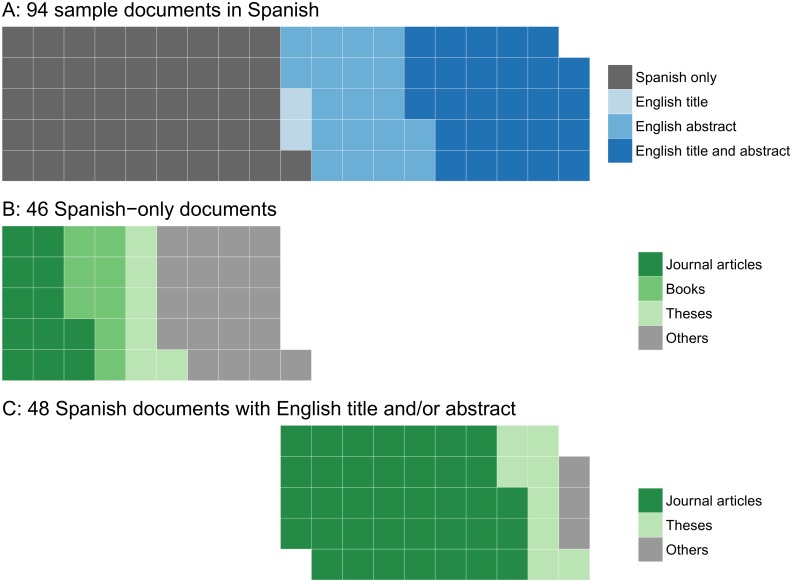
Waffle plots of (A) the use of an English title and an English abstract in 94 scientific documents written in Spanish (sampled from the 9,520 documents searched in Fig 1); document types of (B) the 46 documents with neither an English title nor an English abstract and (C) those with an English title and/or an English abstract. Each square represents one document.

**Fig 3:**
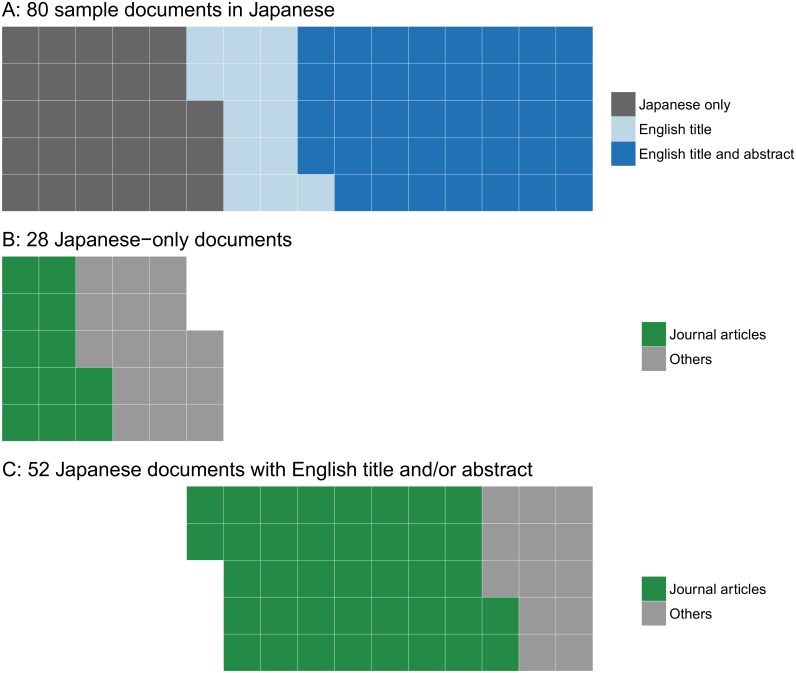
Waffle plots of (A) the use of an English title and an English abstract in 80 scientific documents written in Japanese (sampled from the 474 documents searched in Fig 1); document types of (B) the 28 documents with neither an English title nor an English abstract and (C) those with an English title and/or an English abstract. Each square represents one document.

In fact, the consequences of ignoring non-English science may be more serious than merely lacking access to 36% of existing information; it can cause biases and gaps in our understanding of the global environment. One potential bias in systematic reviews of English-language journals is the over-representation of positive and/or statistically significant results [7], as they are more likely to be published in high-impact English journals. Another type of bias, of particular relevance to environmental sciences, is that information on species, habitats, ecosystems, and phenomena that are specific to countries where English is not the mother tongue can be overlooked when searched only in English, as also reported in medical sciences [5]. As an example, the latest estimates of population status in Taiwan for fairy pittas (*Pitta nympha)*, a bird species of conservation concern, are available only in traditional Chinese (http://www.wracb.gov.tw/public/DownLoads/20157161639457055.pdf) and not used in the global assessment by the International Union for Conservation of Nature (Chie-Jen Ko, personal communications). Similarly, important papers reporting the infection of pigs with avian influenza viruses in China initially went unnoticed by international communities, including the World Health Organization and the United Nations Food and Agriculture Organization, because they were published in Chinese-language journals [8]. Also, there is a recognized knowledge gap about the effects on biodiversity of some crops, such as soybeans, sorghum, and cotton [9], but considering that these crops are grown over large areas in South America and China, scientific literature on these crops may exist in the local languages of these regions. Finally, scientific knowledge generated by those undertaking conservation activities in the field (field practitioners) could also be under-represented in English, as field practitioners often find it a challenge to have their work published in academic journals [4], particularly in English if they are non-native English speakers [10]. This potentially renders local and indigenous knowledge unavailable in English. For example, Wetlands International Argentina has produced over 20 technical publications on the conservation and management of wetlands over the past 20 years, but only 2 are available in English (Daniel E. Blanco, personal communications). Their non-English publications include a report on the roles of peatlands, a wetland type of potential global importance, in mitigating climate change impacts (http://lac.wetlands.org/Portals/4/Turberas/Factbook%20Turberas%20de%20TdF%202010.pdf). Such knowledge generated by practitioners is often overlooked as grey literature but forms a vital part of the evidence base [10]. For instance, the IPBES has recently shown that local and indigenous knowledge is a key to understanding the conservation of ecosystem services by pollinators (http://www.ipbes.net/article/press-release-pollinators-vital-our-food-supply-under-threat).

Another consequence of language barriers that is becoming increasingly important operates in the opposite direction: much scientific knowledge is now unavailable in local languages, as publication in English has become prevalent. A factor behind this is that even scientists whose mother tongue is not English aim to produce papers in English for publication in high-impact journals given the clear advantages for their careers [11]. Furthermore, many journals, previously published in local languages, are now publishing mainly in English to increase their impacts on scientific communities globally (e.g., *Animal Biodiversity and Conservation* in Spain, *Natureza & Conservação* in Brazil). As a consequence, there exists an imbalance in knowledge transfer in countries where English is not the mother tongue; much scientific knowledge that has originated there and elsewhere is available only in English and not in their local languages.

The increase in the proportion of conservation-related papers published in English has helped global English-speaking communities access a broader range of information but, at the same time, potentially raised the barrier for local practitioners and policy makers whose mother tongue is not English. Leaving this problem unresolved is untenable if we consider that areas experiencing a rapid loss of biodiversity and thus in the greatest need of information, education, and conservation practices are often places where English is not spoken widely [12]. The last decade has seen an explosion of papers urging conservation communities to tackle research-implementation gaps (e.g., [13]), but language barriers can further widen these gaps. Conservation science needs to deliver local-level, species-specific evidence to on-site practitioners and policy makers, but many practitioners often find language a barrier when accessing primary scientific information [4, 11]. For example, our survey with 44 national and regional protected areas in Spain revealed that 54% of the directors (13 out of the 24 who responded to our survey) identified languages as a barrier to the use of scientific papers as an information source for management. Thus, although the extent of such language barriers should vary among countries and individuals, depending on their proficiency in English, simply providing scientific knowledge in easily understandable and accessible ways, but in English, might not make a difference for many practitioners and policy makers.

Transcending language barriers requires societal, institutional, and individual-level changes. We should not assume that all important information is available in English. When conducting systematic reviews or developing databases at a global scale, one simple, yet rarely adopted, solution would be to include in the discussion speakers of a wide range of languages (e.g., at least Spanish, Portuguese, Chinese, and French, which, in theory, altogether cover the vast majority of non-English scientific documents; Fig 1). Particularly in influential global assessments, like those by the IPBES, scientific literature published in non-English languages should be equally considered and, if appropriate, included. We obviously need to ensure the quality of literature to be included in such assessments; involving native speaker(s) of each language would also facilitate this process. To this end, the website ConservationEvidence.com is establishing an international panel to extract non-English language papers on conservation interventions. In situations in which this approach is impractical, the use of non-English search terms would help identify relevant non-English literature, although it is still not a common practice. We also suggest developing a database of major non-English journals in the discipline (a partial list is shown in S1 Table for conservation science). Such a database can be accompanied by the registration of investigators working on a particular topic so that even nonindexed works can be shared, as suggested and implemented in medical sciences [5, 14], and relevant papers can be disseminated in English via, for example, Social Network Services. Authors of non-English language papers could also try to increase the visibility of their papers by uploading preprints or postprints with the titles and abstracts in English on well-recognized online repositories (see below).

A key to facilitating the application of scientific knowledge expressed in English to local environmental issues is multilingualization of the knowledge. While English plays a crucial role in the current publishing systems by centralizing scientific knowledge, we also need a system for effectively redistributing the compiled knowledge to its users. We propose that all authors be requested to provide lay summaries when publishing their papers in relevant conservation journals. The journals could then provide translations of those summaries in multiple languages. This would dramatically increase accessibility to scientific knowledge for practitioners and policy makers, as knowledge is provided regularly at a specific location(s) in an easily understandable way. It would be even more influential if major journals in the subject area could establish a common website. Translation costs could be covered by either journals or authors depending on funding availability, in the same way that several open access journals offer full or partial waivers to overcome any financial barriers to publication. Another, though less influential, approach is to encourage individual researchers to provide translations of their papers, for example, as supporting information of the original English papers (*PLOS* journals and *Conservation Biology* allow this [15, 16]; see S1–S5 Abstracts for the lay summary of this paper in Spanish, Portuguese, French, simplified Chinese, and Japanese) or through self-archiving on institutional or other repositories under appropriate copyright conditions. For instance, submissions in multiple languages and translations of previously published work are accepted in arXiv (https://arxiv.org/help/faq/multilang, http://arxiv.org/help/translations), figshare (confirmed on 23 February 2016), and PeerJ (confirmed on 15 March 2016). For the translation of scientific books, a successful business model has already been proposed [12], which could be adopted widely.

While outreach activities have recently been advocated in science, it is still rare for such activities to involve communication across language barriers. Institutions could give credit to efforts by researchers to translate their findings into local languages in a similar way to how other outreach activities are evaluated, particularly if the research covers issues at the global scale or regions where English is not the mother tongue. Funding bodies and societies can encourage researchers to use their funding for multilingualization; plans to overcome language barriers, where appropriate, can be a criterion for evaluating outreach activities in grant proposals (e.g., the British Ecological Society’s Outreach Grants and the National Science Foundation’s Broader Impacts Review Criterion). As facilitating the translation of English knowledge to a local language can benefit the local community, this could also attract the attention of local funders [12].

Language barriers continue to impede the global compilation and application of scientific knowledge. Overcoming this problem is not an easy challenge, but when achieved should have far-reaching benefits to both scientists and users of scientific information in tackling global environmental changes and solving local environmental issues. We believe the approaches described here offer potential practical solutions.

## Supporting Information

S1 Table Number of scientific documents in 16 languages. (DOCX)

S1 Abstract Alternative Language Abstract in Spanish. (DOCX)

S2 Abstract Alternative Language Abstract in Portuguese. (DOCX)

S3 Abstract Alternative Language Abstract in French. (DOCX)

S4 Abstract Alternative Language Abstract in simplified Chinese. (DOCX)

S5 Abstract Alternative Language Abstract in Japanese. (DOCX)

## Abbreviations

IPBES: Intergovernmental Platform on Biodiversity and Ecosystem Services
