# Supplementary material for: Languages Are Still a Major Barrier to Global Science
Source: PLoS Biol. 2016 Dec 29;14(12):e2000933. doi: 10.1371/journal.pbio.2000933 (PMC5199034; doi:10.1371/journal.pbio.2000933)
Supplement: S1 Table — (DOCX) [file pbio.2000933.s001.docx]

**Supporting Information**

**Table S1.** The number of scientific documents (excluding patents and citations) in 2014 alone based on a search with two keywords “biodiversity” and “conservation” in 16 major languages on Google Scholar. Each search was restricted only to pages written in the relevant language (apart from Swedish, Russian and Persian, for which this search option was not available). The chosen 16 languages are the national languages of the 20 highest ranked countries in the World Bank’s indicator for scientific and technical journal articles in 2009 (http://data.worldbank.org/indicator/IP.JRN.ARTC.SC). Note that journal examples do not include those that focus on fundamental ecology, which often publish papers on conservation.

| Language | Number of documents  (key words) | Date of search | Examples of peer-reviewed journals on biodiversity conservation (location of publication, number of papers in 2014) |
| --- | --- | --- | --- |
| English | 48,600  (“biodiversity” “conservation”) | 2/2/2016 | Conservation Biology (US, 181)  Conservation Letters (UK, 74)  Biological Conservation (UK, 326) |
| Spanish | 9,520  (“biodiversidad” “conservación”) | 2/2/2016 | Ecosistemas (*Ecosystems*: Spain, 49)  Revista Mexican de Biodiversidad (*Mexican Journal of Biodiversity:* Mexico, 198)  Revista Caldasia (*Caldasia Journal*: Colombia, 29) |
| Portuguese | 7,800  ("biodiversidade" "conservação") | 2/2/2016 |  |
| Chinese (Simplified) | 4,540  (“生物多样性” “保护”) | 2/2/2016 | 应用生态学报 (*Chinese Journal of Applied Ecology*: China, 482)  生物多样性 (*Biodiversity Science*: China, 110) |
| French | 2,290  ("biodiversité" "conservation") | 2/2/2016 |  |
| Italian | 720  ("biodiversità" "conservazione") | 2/2/2016 | Biologia Ambientale (*Environmental Biology*: Italy, 21) |
| German | 601  ("biodiversität" "naturschutz")^1^ | 2/2/2016 |  |
| Japanese | 474  (“生物多様性” “保全”) | 2/2/2016 | 保全生態学研究 (*Japanese Journal of Conservation Ecology*: Japan, 19) |
| Korean | 207  ("생물 다양성" "보전") | 2/2/2016 | 한국자연보호학회지 (*Korean Journal of Nature Conservation*: South Korea, 17)  한국환경생태학회지 (*Korean Journal of Environment and Ecology*: South Korea, 79) |
| Swedish | 184  ("biologisk mångfald" "bevarande") | 2/2/2016 |  |
| Chinese  (Traditional) | 136  ("生物多樣性" "保育") | 2/2/2016 | 台灣生物多樣性研究 (*Taiwan Journal of Biodiversity*: Taiwan, 26)^2^ |
| Polish | 131  ("bioróżnorodność" "ochrona")^3^ | 2/2/2016 | Chrońmy Przyrodę Ojczystą (*Let’s Protect Our Indigenous Nature*: Poland, 77)  Kulon (*Curlew*: Poland, 20)  Przegląd Przyrodniczy (*Nature Review*: Poland, 23) |
| Turkish | 124  ("biyolojik çeşitlilik" "koruma")^4^ | 2/2/2016 |  |
| Russian | 82  ("биоразнообразие" "охрана природы") | 2/2/2016 |  |
| Persian | 55  ("حفاظت" "تنوع زیستی") | 2/2/2016 | نشریه محیط زیست طبیعی (*Journal of Natural Environment*: Iran, 40)  مجله بوم شناسی کاربردی (*Iranian* *Journal of Applied Ecology*: Iran, 35) |
| Dutch | 49  ("biodiversiteit" "natuurbehoud") | 2/2/2016 | De Levende Natuur (*The Living Nature*: Netherlands, 41) |

^1^"biodiversitaet" and "artenvielfalt" for biodiversity, and "umweltschutz" and "artenschutz" for conservation were also used and the combination with the highest number of documents is presented.

^2^Only non-English papers are included (four papers published in 2014 are in English)

^3^"różnorodność biotyczna" for biodiversity, and “konserwacja” for conservation were also used and the combination with the highest number of documents is presented.

^4^"biyoçeşitlilik" was also used for biodiversity but not presented as it returned a smaller number of documents.
