# Supplementary material for: Languages Are Still a Major Barrier to Global Science
Source: PLoS Biol. 2016 Dec 29;14(12):e2000933. doi: 10.1371/journal.pbio.2000933 (PMC5199034; doi:10.1371/journal.pbio.2000933)
Supplement: S1 Abstract — (DOCX) [file pbio.2000933.s002.docx]

**Las barreras lingüísticas siguen obstaculizando la ciencia**

Tatsuya Amano*, Juan P. González-Varo, William J. Sutherland

Conservation Science Group, Department of Zoology, University of Cambridge, The David Attenborough Building, Pembroke Street, Cambridge CB2 3QZ, UK.

* amatatsu830@gmail.com

El inglés es indudablemente a día de hoy el idioma más ampliamente utilizado en las actividades científicas globales, tales como la publicación de artículos y la presentación de investigaciones en conferencias. Por este motivo, asumimos con frecuencia que todo conocimiento científico relevante está disponible y comunicable en inglés. Pero ¿Es esto cierto?

Creemos que la respuesta es no, según los resultados de nuestro trabajo "Los idiomas siguen siendo una barrera importante para la ciencia global", publicado en PLOS Biology. Creemos que las barreras lingüísticas son particularmente un serio problema en las ciencias ambientales. De hecho, hay un reconocimiento creciente de que la compilación de conocimientos científicos globales (p.ej. evidencia sobre la efectividad de las intervenciones con fines de conservación) es crucial para proporcionar información relevante en la toma de decisiones políticas y en las prácticas ambientales. Este trabajo intenta entender cómo las barreras del lenguaje pueden obstaculizar estos dos procesos de transferencia de conocimiento.

Primero estimamos el número de documentos científicos escritos en los 16 idiomas principales del mundo buscando en Google Académico las palabras clave "biodiversidad" y "conservación" traducidas en estos idiomas. El resultado fue bastante sorprendente: encontramos que de un total de 75.513 documentos publicados en 2014, sólo el 64% estaba en inglés. El resto estaba escrito en los otros 15 idiomas, principalmente en español (13%), portugués (10%), chino simplificado (6%) y en francés (3%). Así, en teoría, centrarse sólo en la ciencia escrita en inglés podría omitir el 36% de los conocimientos existentes.

Podrías aún pensar que el conocimiento importante se publica en gran medida inglés. Sin embargo, ignorar la ciencia no inglesa puede causar sesgos y lagunas en nuestra comprensión del ambiente global, porque (i) los resultados positivos y estadísticamente significativos tienden publicarse en revistas inglesas, (ii) la información sobre especies, hábitats y ecosistemas en países de habla no inglesa puede pasarse por alto cuando se busca sólo en inglés, y (iii) el conocimiento generado por los gestores a menudo está disponible sólo en idiomas locales.

También examinamos otra consecuencia de las barreras lingüísticas: muchos conocimientos científicos no están disponibles en los idiomas locales, ya que la publicación en inglés se ha vuelto prevalente. Esto plantea potencialmente una barrera para los gestores locales y responsables políticos que tratan de acceder al conocimiento científico, si sus habilidades en inglés son limitadas. Esta idea fue apoyada por nuestra encuesta a 44 áreas protegidas en España, donde encontramos que el 54% de los directores de conservación (13 de 24 que respondieron) identificaron el idioma como una barrera en el uso del conocimiento científico para la gestión.

¿Cómo podemos resolver este problema entonces? Proporcionamos una serie de sugerencias. Las soluciones para compilar conocimiento no-inglés incluyen: involucrar a nativos de otros idiomas y usar palabras clave no inglesas en las búsquedas bibliográficas, así como incrementar la visibilidad de la bibliografía no inglesa a través del desarrollo de una base de datos para revistas no inglesas y el uso de repositorios *online* acreditados. Para facilitar la multilingüización del conocimiento actual disponible actualmente en inglés, proponemos la publicación de en las páginas web de las revistas de resúmenes divulgativos traducidos y asociados a artículos científicos relevantes para la conservación (tal y como hemos hecho con este documento). También, la publicación de traducciones completas de los artículos originales y su publicación *online* como material suplementario.

Superar las barreras idiomáticas para la ciencia no es un reto fácil. Sin embargo, superar tales barreras debe tener amplios beneficios, tanto para los científicos como para los usuarios de la información científica, a la hora de abordar los cambios ambientales globales y resolver los problemas ambientales locales. Creemos que los enfoques aquí descritos ofrecen soluciones prácticas factibles.
