# Supplementary material for: Languages Are Still a Major Barrier to Global Science
Source: PLoS Biol. 2016 Dec 29;14(12):e2000933. doi: 10.1371/journal.pbio.2000933 (PMC5199034; doi:10.1371/journal.pbio.2000933)
Supplement: S2 Abstract — (DOCX) [file pbio.2000933.s003.docx]

**As barreiras linguísticas continuam a ser um obstáculo para a ciência**

Tatsuya Amano*, Juan P. González-Varo, William J. Sutherland

Conservation Science Group, Department of Zoology, University of Cambridge, The David Attenborough Building, Pembroke Street, Cambridge CB2 3QZ, UK.

* amatatsu830@gmail.com

traduzido por Ana Jesus

O inglês é, sem dúvida, o idioma mais utilizado hoje em ciência a nível global, incluindo na publicação de artigos científicos e apresentação de projetos de investigação em conferências. Por esta razão, muitas vezes assumimos que todo o conhecimento científico importante está disponível, e é comunicado, em Inglês. Mas será verdade?

Acreditamos que não, com base nos resultados do nosso artigo “Languages are still a major barrier to global science”, publicado em PLOS Biology. Na nossa opinião, as barreiras linguísticas são um problema sério, particularmente nas ciências ambientais.

Cada vez mais se reconhece que a compilação de conhecimento científico a nível global (por exemplo, sobre a eficácia das práticas de conservação), e torná-lo acessível é fundamental para melhorar as políticas e práticas ambientais. Este artigo procura entender de que forma as barreiras da língua impedem estes dois processos de transferência de conhecimento.

Primeiro, estimamos o número de publicações científicas escritas nos 16 idiomas principais pesquisando no *Google Scholar* as duas palavras-chave "biodiversidade" e "conservação" nesses 16 idiomas.

O resultado foi impressionante. Descobrimos que de um total de 75,513 documentos publicados em 2014, apenas 64% estava em inglês. Os restantes estavam escritos noutros 15 idiomas, como o espanhol (13%), português (10%), chinês simplificado (6%) e francês (3%). Como tal, teoricamente, a utilização da ciência escrita apenas em inglês pode impedir o acesso a 36% do conhecimento existente.

Talvez pense que o conhecimento importante é na maioria publicado em Inglês. Mas na verdade, ignorar a ciência publicada noutros idiomas pode gerar percepções incorretas e lacunas no nosso entendimento do ambiente a nível global. Isto porque (i) resultados positivos e estatisticamente significativos tendem a ser publicados em revistas de língua inglesa, (ii) informação sobre espécies, habitats e ecossistemas em países onde o inglês não é a língua materna tende a ser negligenciada quando pesquisada apenas em inglês, e (iii) o conhecimento gerado por profissionais encontra-se muitas vezes apenas disponível no idioma local.

Observamos também outra consequência das barreiras linguísticas: uma grande parte do conhecimento científico não se encontra disponível nos idiomas locais, já que publicar em inglês se tornou prevalente. O que potencialmente cria uma barreira para profissionais e decisores políticos locais que tentem aceder a conhecimento científico, com pouco domínio do inglês. Como demonstra o estudo que efetuamos em 44 áreas protegidas de Espanha, onde 54% dos diretores (13 dos 24 que responderam) identificaram a língua como uma barreira ao uso do conhecimento científico para a gestão ambiental.

Como é que podemos resolver este problema? Propomos várias sugestões. Abordagens para compilar conhecimento disponível noutros idiomas incluem: envolver nativos e usar palavras-chave noutros idiomas em pesquisas bibliográficas, aumentar a visibilidade da literatura não inglesa através do desenvolvimento de um base de dados para revistas científicas noutros idiomas, e a utilização de repositórios *online* prestigiados.

Para facilitar a divulgação do conhecimento científico disponível somente em inglês, propomos que as revistas científicas publiquem nas suas páginas *web*, como material suplementar, resumos não técnicos em vários idiomas (como tentámos fazer com este documento), assim como traduções completas de artigos originais de relevância para a conservação da natureza.

Superar as barreiras da língua em ciência não é um desafio fácil, mas quando alcançado terá certamente grandes benefícios tanto para os cientistas como para aqueles que utilizam a informação científica para lidar com mudanças ambientais a nível global e resolver questões ambientais de carácter local. Acreditamos que as propostas aqui descritas oferecem possíveis soluções práticas nesse sentido.
