# Supplementary material for: Languages Are Still a Major Barrier to Global Science
Source: PLoS Biol. 2016 Dec 29;14(12):e2000933. doi: 10.1371/journal.pbio.2000933 (PMC5199034; doi:10.1371/journal.pbio.2000933)
Supplement: S3 Abstract — (DOCX) [file pbio.2000933.s004.docx]

**Les barrières linguistiques continuent d’entraver les avancées scientifiques**

Tatsuya Amano*, Juan P. González-Varo, William J. Sutherland

Conservation Science Group, Department of Zoology, University of Cambridge, The David Attenborough Building, Pembroke Street, Cambridge CB2 3QZ, UK.

* amatatsu830@gmail.com

Traduit par Alice Poirier

L'anglais est sans aucun doute la langue la plus utilisée aujourd'hui dans les activités scientifiques à l’échelle mondiale, y compris la publication d’articles scientifiques et la présentation de travail de recherche à des conférences. De ce fait, nous supposons souvent que toutes les connaissances scientifiques importantes sont disponibles, et communicables, en anglais. Mais est-ce bien vrai ?

Nous sommes d’avis que la réponse est non, basée sur les conclusions de notre article "Les langues sont encore un obstacle majeur à l’avancée des sciences à l’échelle mondiale", publié dans PLOS Biology. Nous pensons que les barrières linguistiques constituent un grave problème, en particulier dans le domaine des sciences de l'environnement. Il est de plus en plus reconnu que compiler les connaissances scientifiques mondiales (par exemple comme preuve testant l'efficacité d’interventions de conservation), et les rendre largement accessibles, est crucial pour mieux informer les politiques et pratiques environnementales. Cet article tente de comprendre comment les barrières linguistiques peuvent entraver ces deux processus de transfert de connaissances.

Nous avons d'abord estimé le nombre de documents scientifiques écrits dans les 16 principales langues du monde en recherchant sur Google Scholar les deux mots-clés «biodiversité» et «conservation» dans ces 16 langues. Le résultat a été assez frappant ; Nous avons constaté que sur un total de 75 513 documents publiés en 2014, seulement 64% étaient en anglais. Le reste était écrit dans les 15 autres langues, telles que l'espagnol (13%), le portugais (10%), le chinois simplifié (6%) et le français (3%). Aussi qu’en théorie, se concentrer uniquement sur le travail scientifique écrit en anglais pourrait faire perdre l'accès à 36% des connaissances existantes.

Vous pouvez continuer de penser que les connaissances importantes sont largement publiées en anglais. En réalité, ignorer la communication scientifique non anglaise peut causer des biais et des lacunes dans notre compréhension de l'environnement global, car (i) les résultats positifs et statistiquement significatifs tendent à être publiés dans des revues anglaises, (ii) les informations sur les espèces, les habitats et les écosystèmes dans les pays où l'anglais n'est pas la langue maternelle peuvent être négligées lorsqu'elles sont recherchées uniquement en anglais, et (iii) les connaissances produites par les praticiens sont souvent disponibles uniquement dans les langues locales.

Nous avons également examiné une autre conséquence des barrières linguistiques : beaucoup de connaissances scientifiques sont maintenant indisponibles dans les langues locales car la publication en anglais est devenue courante. Cela peut potentiellement entraver les praticiens locaux et les responsables politiques qui cherchent à accéder aux connaissances scientifiques, si leurs compétences en anglais ne sont pas suffisantes. Cette idée a été soutenue par notre enquête sur 44 aires protégées en Espagne, où nous avons constaté que 54% des directeurs (13 sur 24 qui ont répondu) identifiaient la langue comme un obstacle à l'utilisation des connaissances scientifiques pour la gestion.

Comment alors pouvons-nous résoudre ce problème ? Nous fournissons un certain nombre de suggestions. Les approches servant à compiler des connaissances autres que l'anglais comprennent : l’implication de locuteurs natifs de langues autres que l'anglais et l'utilisation de mots-clés non anglais dans les recherches documentaires, ainsi que l'accroissement de la visibilité de la littérature non anglaise par le développement d'une base de données pour des revues non anglaises et l’emploi de référentiels en ligne bien reconnus. Afin de faciliter le multilinguisme des connaissances actuellement disponibles en anglais, nous proposons que les traductions des articles publiés soient disponibles sous forme de résumés amateurs sur les sites Web des revues (comme nous l'avons tenté avec ce document), ou bien d'informations complémentaires, de prépublications et de post-impressions des documents originaux.

Surmonter les barrières linguistiques dans les sciences n'est pas un défi facile, qui néanmoins lorsqu'il est atteint devrait profondément bénéficier tant les scientifiques que les utilisateurs de l'information scientifique, afin d’aborder les changements environnementaux à l’échelle mondiale et de résoudre les problèmes environnementaux locaux. Nous croyons que les approches décrites ici offrent des solutions pratiques potentielles.
