# Supplementary material for: Languages Are Still a Major Barrier to Global Science
Source: PLoS Biol. 2016 Dec 29;14(12):e2000933. doi: 10.1371/journal.pbio.2000933 (PMC5199034; doi:10.1371/journal.pbio.2000933)
Supplement: S4 Abstract — (DOCX) [file pbio.2000933.s005.docx]

**语言障碍继续阻碍科学交流**

Tatsuya Amano*, Juan P. González-Varo, William J. Sutherland

剑桥大学动物学系 大卫阿滕伯勒楼，彭布罗克街，剑桥， CB2 3QZ, UK

* amatatsu830@gmail.com

Translated by Dr. Min Chen

现在，国际上发表论文和会议报告等科学信息交流中最普遍使用的语言无疑是英语。我们常认为所有重要的科学知识都可通过英语获得和沟通。但这是真的吗？

我们认为答案是“不”。根据我们发表在PLOS Biology的论文中的调查结果发现：语言仍然是全球科学交流的主要障碍，尤其在环境科学领域。人们越来越认识到汇编全球范围检验环境保护措施有效性的实证研究，并将有数据支持的有效保护措施推广到环境方面的政策与实践的重要性。本文将从两方面来揭示语言障碍的表现。

我们首先利用16种世界主要语言对关键词“生物多样性”和“保护”的表达在谷歌学术搜素，通过该结果来估计世界上的使用这16种语言编写的相关科学文献数量分布。得到了惊人的数据：在2014年发表的75513篇相关文献中只有64%是用英语写作的，其余的由西班牙语（13%），葡萄牙语（10%），简体中文（6%）和法语（3%）等15种语言组成。因此，理论上只关注英语著作可能会丢失36%的现有知识。

你可能仍然认为重要的知识大部分是用英语发表的。但事实上，忽视非英语的科学文献会导致我们对全球环境的认识产生偏差和漏洞，因为(i)正向的和统计上显著的结果倾向于在英文期刊发表，（ii）只使用英语搜索时会忽略非英语国家物种、生境及生态系统的信息，（iii）从业者总结的经验往往只用当地语言来表达。

我们还研究了语言障碍带来的另一个结果：现在许多科学知识普遍用英语发表而不是当地语言。对于英语不好的从业者和政策制定者，这可能会增加他们获得科学经验的难度。这个结果也得到了我们的调查数据的支持：在西班牙的44个保护区中54%的负责人（24人中13人给予反馈）认为搜寻有效的科学管理知识时存在语言障碍。

那么我们如何才能解决这个问题呢？我们建议汇编非英语知识：非英语母语的人用其母语关键词在其母语数据库和认知度好的在线报道中进行搜索，然后将这些多元的语言转换成英语。我们建议翻译可利用的非英语论文的摘要或原始论文预印本及后印本中支持信息，然后在期刊网站上公布（我们已试运行）。

克服科学领域的语言障碍是项艰巨的挑战。我们将开展工作帮助科学家和使用者摆脱语言障碍，获得和更新解决全球和地方环境问题的有效方法。我们相信本文所述的研究将是实际解决语言障碍的有效途径。
