# Supplementary material for: Languages Are Still a Major Barrier to Global Science
Source: PLoS Biol. 2016 Dec 29;14(12):e2000933. doi: 10.1371/journal.pbio.2000933 (PMC5199034; doi:10.1371/journal.pbio.2000933)
Supplement: S5 Abstract — (DOCX) [file pbio.2000933.s006.docx]

**科学の発展を妨げ続ける言語による障壁**

天野達也*, Juan P. González-Varo, William J. Sutherland

ケンブリッジ大学　動物学部　保全科学グループ, The David Attenborough Building, Pembroke Street, Cambridge CB2 3QZ, UK.

* amatatsu830@gmail.com

現在、論文出版や学会発表など幅広い科学的活動において、英語は世界で最も用いられている言語として重要な役割を果たしています。そのため、しばしば重要な科学的知見はすべて英語で手にすることができ、また英語で人々に伝えることができると考えられがちですが、これは果たして本当なのでしょうか？

PLOS Biologyに出版された本論文“Languages are still a major barrier to global science” は、そんな認識が誤っている可能性を示しています。特に環境科学では、対策の有効性を検証した科学的知見を国際的に集約し、その知見を各地域での環境政策や保全活動につなげていくことが重要であるため、その過程で言語による「障壁」が重大な帰結をもたらすと考えられます。本論文では、科学的知見を集約・伝達する過程で、言語の障壁がどのような影響を及ぼしうるのか考察を行いました。

本論文ではまず初めに、「生物多様性」と「保全」という二つのキーワードを用いたGoogle Scholarでの文献検索によって、世界の主要16言語それぞれでどのくらいの科学文書が出版されているのかを調べました。その結果、2014年に出版された75,513文書のうち、英語の文書が占める割合は64％であることが分かりました。残りの36％は他の15言語によって書かれており、特にスペイン語（13％）、ポルトガル語（10％）、簡体字中国語（6％）、フランス語（3％）による文書が多くの割合を占めていました。この結果は、英語のみに注目することで36％もの既存の科学的知見を見失ってしまう可能性を示しています。

それでも多くの人は、重要な科学的知見は英語で出版されていると考えがちです。しかし実際には、こういった英語以外の言語で出版されている科学的知見を見過ごすことで、世界の環境について偏った理解を生み出す可能性があると考えられます。その理由として、（i）肯定的な結果や統計的に有意な結果が偏って多く英語で出版されやすいこと、（ii）英語が母語でない国に特異的な種や生息地、生態系などに関する情報はその国の言語で出版されやすいこと、（iii）保全従事者によって得られた知見は英語では出版されにくいこと、などが挙げられます。

本論文では言語による障壁がもたらすもう一つの帰結、すなわち、近年ますます多くの論文が英語で出版されるため、重要な知見が英語以外の言語では得にくい、という問題についても議論しています。その結果、英語が母語でない多くの保全従事者や政策決定者にとって、科学的知見を利用する際に英語が大きな障壁となっています。例えば、スペインの44自然保護区を対象としたアンケート調査によって、54％（回答のあった24保護区のうち13）の保護区では、科学的知見を保全管理に利用する際に英語が障壁となっているということが分かりました。

ではどうすればこの問題を解決できるのでしょうか？本論文ではその解決策についてもいくつか提案をしています。例えば英語以外の言語による科学的知見を効率よく収集するためには、研究プロジェクトに様々な言語の話者を含める、文献検索で英語以外の言語のキーワードを用いる、英語以外の言語の主要学術雑誌を網羅したデータベース構築や、国際的に認知されたレポジトリの活用によって、非英語の文献を国際的に認知されやすくする、といった対策が考えられます。一方で、現在は英語のみで得られる科学的知見を他の言語でも得られるようにするためには、英語で出版される論文の概要を複数言語で学術雑誌のウェブサイト上に公開する（この文章はその試みの一環です）、また論文の翻訳を付録やプレプリント、ポストプリントとして公開する、といった対策が考えられます。

科学における言語の障壁を克服していくことは簡単ではありません。しかしながら、もしこの問題を克服することができれば、科学者のみならず科学的知見を利用する誰もにとって、地球規模の環境変化という問題、そして地域的な環境問題に取り組む際に、大きな恩恵があると考えられます。ここで提案した対策がそのための一助となれば幸いです。
